# Validation of Lung EpiCheck, a novel methylation-based blood assay, for the detection of lung cancer in European and Chinese high-risk individuals

**DOI:** 10.1183/13993003.02682-2020

**Published:** 2021-01-14

**Authors:** Mina Gaga, Joanna Chorostowska-Wynimko, Ildikó Horváth, Martin C. Tammemagi, David Shitrit, Vered H. Eisenberg, Hao Liang, David Stav, Dan Levy Faber, Maarten Jansen, Yael Raviv, Vasileios Panagoulias, Piotr Rudzinski, Gabriel Izbicki, Ohad Ronen, Adiv Goldhaber, Rawia Moalem, Nadir Arber, Ilana Haas, Qinghua Zhou

**Affiliations:** 1 7th Respiratory Medicine Dept, Athens Chest Hospital, Athens, Greece; 2 National Institute of Tuberculosis and Lung Diseases, Warsaw, Poland; 3 National Korányi Institute of Pulmonology, Budapest, Hungary; 4 Brock University, St Catharines, ON, Canada; 5 Pulmonary Dept, Meir Medical Center, Kfar Saba, Israel; 6 Sackler Faculty of Medicine, Tel Aviv University, Tel Aviv, Israel; 7 Lung Cancer Center/Lung Cancer Institute, West China Hospital, Sichuan University, Chengdu, China; 8 Lung Institute, Maccabi Health Services Hashalom, Tel-Aviv, Israel; 9 Dept of Cardiothoracic Surgery, Lady Davis Carmel Medical Center, Haifa, Israel; 10 Ruth and Bruce Rappaport Faculty of Medicine, Technion – Israel Institute of Technology, Haifa, Israel; 11 Pulmonary Dept, Ziekenhuisgroep Twente, Hengelo, The Netherlands; 12 Dept of Medicine, Pulmonology Institute, Soroka Medical Center, Ben-Gurion University, Beer-Sheva, Israel; 13 2nd Respiratory Medicine Dept, Athens Chest Hospital, Athens, Greece; 14 Pulmonary Institute, Shaare Zedek Medical Center, Jerusalem, Israel; 15 Dept of Otolaryngology – Head and Neck Surgery, Galilee Medical Center affiliated with Azrieli Faculty of Medicine, Safed, Israel; 16 Clalit Health Services, Raanana, Israel; 17 Gastroenterology Institute, The Holy Family Hospital, Nazareth, Israel; 18 Integrated Cancer Prevention Center, Tel Aviv Sourasky Medical Centre, Sackler Faculty of Medicine, Tel Aviv University, Tel-Aviv, Israel; 19 Breast Unit, Meir Medical Center, Kfar Saba, Israel

## Abstract

**Aim:**

Lung cancer screening reduces mortality. We aim to validate the performance of Lung EpiCheck, a six-marker panel methylation-based plasma test, in the detection of lung cancer in European and Chinese samples.

**Methods:**

A case–control European training set (n=102 lung cancer cases, n=265 controls) was used to define the panel and algorithm. Two cut-offs were selected, low cut-off (LCO) for high sensitivity and high cut-off (HCO) for high specificity. The performance was validated in case–control European and Chinese validation sets (cases/controls 179/137 and 30/15, respectively).

**Results:**

The European and Chinese validation sets achieved AUCs of 0.882 and 0.899, respectively. The sensitivities/specificities with LCO were 87.2%/64.2% and 76.7%/93.3%, and with HCO they were 74.3%/90.5% and 56.7%/100.0%, respectively. Stage I nonsmall cell lung cancer (NSCLC) sensitivity in European and Chinese samples with LCO was 78.4% and 70.0% and with HCO was 62.2% and 30.0%, respectively. Small cell lung cancer (SCLC) was represented only in the European set and sensitivities with LCO and HCO were 100.0% and 93.3%, respectively. In multivariable analyses of the European validation set, the assay's ability to predict lung cancer was independent of established risk factors (age, smoking, COPD), and overall AUC was 0.942.

**Conclusions:**

Lung EpiCheck demonstrated strong performance in lung cancer prediction in case–control European and Chinese samples, detecting high proportions of early-stage NSCLC and SCLC and significantly improving predictive accuracy when added to established risk factors. Prospective studies are required to confirm these findings. Utilising such a simple and inexpensive blood test has the potential to improve compliance and broaden access to screening for at-risk populations.

## Abstract 2

**Lung EpiCheck, a simple blood test, detected 85% of early-stage lung cancers with specificity of 64% in high-risk population, reaching AUC of 0.942 when combined with risk factors. This could improve efficiency of implementing lung cancer screening.** https://bit.ly/3jWhLOn

## Introduction

Lung cancer is the leading cause of death from cancer, with 1.76 million deaths worldwide in 2018 [1]. Risk factors include age, smoking, family history and occupational/asbestos exposure. 5-year survival rate for lung cancer is only 18.6%, mainly due to diagnosis at late stages [2]. Screening with low-dose computed tomography (LDCT) has been proven to reduce lung cancer mortality in high-risk populations [3, 4]. However, LDCT has a significant rate of false positives and overdiagnosis, involves radiation hazard, is reader dependent and requires substantial infrastructure. In the USA, up to 14% of the eligible population undergo lung cancer screening [5]. Current barriers are infrastructure and knowledge and awareness gaps among referring physicians and the public. Importantly, lung cancer screening is targeting a very high-risk population, representing merely a quarter of lung cancer patients [6].

Several types of tumour-derived biomarkers have been assessed for lung cancer detection, including circulating tumour cells, exosomes, mutations and methylation changes in cell-free (cf)DNA, microRNA and proteins [7, 8]. Genome-wide hypomethylation and hypermethylation changes are found in lung cancer and could potentially serve as markers [9].

EpiCheck is a simple ultrasensitive PCR-based assay that detects cancer-associated hypermethylation changes in a selected panel of markers from any body fluid or tissue. The urine-based Bladder EpiCheck demonstrated sensitivity of 92% for high-grade urothelial carcinoma with specificity of 88% in bladder cancer patients undergoing surveillance [10].

The purpose of this study is to validate the performance of Lung EpiCheck®, a six-methylation-marker blood test, in lung cancer detection.

## Methods

### Study samples

Training set samples were used to select the markers for the panel using Nucleix's proprietary bioinformatics techniques (Nucleix, Rehovot, Israel). Six markers were selected based on their synergistic information and an algorithm calculating the EpiScore was developed and locked down (supplementary figure S1). Two cut-offs were defined to allow for different clinical scenarios, a low cut-off (LCO) of EpiScore ≥60, favouring high sensitivity, and a high cut-off (HCO) of EpiScore ≥70, favouring high specificity. The European validation set was a new set of samples used to validate the performance of the assay using the pre-defined algorithm and cut-offs.

The training and the European validation sets were obtained by applying a single protocol: a case–control study performed on samples from sequential recruitment in 18 departments and clinics in 16 healthcare organisations, and three biobanks in Europe and Israel (supplementary table S2). Samples were collected from July 2016 to March 2018. The initial series of cases and controls were used for training and the subsequent series was used for validation. Cases were recruited from pulmonology, thoracic surgery and oncology departments and clinics in Europe and Israel. Present and past smokers, serving as controls, were recruited from blood collection stations in primary care clinics and from general surgery departments in Israel. Potential participants were randomly approached as they came to perform a blood test for any reason (table 1). Sample processing was performed at the Nucleix laboratory (Rehovot). Disease staging of the cases was according to the American Joint Committee on Cancer staging manual (AJCC)7 and AJCC8. Adenocarcinomas were included only if classified as invasive adenocarcinomas according to International Association for the Study of Lung Cancer/American Thoracic Society/European Respiratory Society classification [11].

**TABLE 1:** Eligibility criteria

	**Inclusion criteria**	**Exclusion criteria**
**Cases**	Subjects with pathologically proven primary lung cancer (NSCLC and SCLC) or Subjects with suspected primary lung cancer undergoing a diagnostic procedure. Patients enrolled with suspicion of lung cancer were included in the analysis if diagnosis was pathologically confirmed primary lung cancer (SCLC and NSCLC)	Subjects with cancer, other than lung cancer Subjects with history of cancer of any kind (except for fully resected nonmelanoma skin cancer)
**Controls**	European sets: Age ≥50 years Current or former smoker Chinese set: Healthy individuals willing to donate blood for the study	Subjects with current diagnosis or history of cancer of any kind (except for fully resected nonmelanoma skin cancer)

NSCLC: nonsmall cell lung cancer; SCLC: small cell lung cancer.

The Chinese validation set was a small feasibility study assessing the applicability of Lung EpiCheck for lung cancer detection in a Chinese population. This was a blinded, case–control, single-centre study performed in the Lung Cancer Center/Lung Cancer Institute at the West China Hospital (Sichuan University, Chengdu, China). Samples were collected from January 2018 to November 2018. Patients suspected or confirmed to have lung cancer arriving for lung surgery were enrolled. Healthy volunteers were enrolled sequentially as controls (table 1). Sample processing was performed on site. Disease staging for the cases was according to AJCC8.

Relevant medical, smoking and family history data were collected prior to study-related procedures. The study was approved by the ethics committees of the various institutions involved, and all subjects provided signed informed consent. The study registration number is NCT02373917.

### Lung EpiCheck testing

Lung EpiCheck (Nucleix) is a blood test that detects lung cancer-associated hypermethylation in six markers in cfDNA. Plasma is separated from a 10 mL EDTA tube within 4 h of blood draw by two consecutive centrifugations at 1500×*g* for 10 min and stored at −20°C to −80°C until DNA extraction. Lung EpiCheck's reagents and methylation-sensitive enzymes are used for DNA extraction, digestion and amplification in real-time PCR (ABI 7500 Fast Dx; Thermo Fisher Scientific, Carlsbad, CA, USA). Three PCR wells are amplified for the markers and one for an internal control to verify the quality of plasma separation by detecting leukocyte-derived DNA. Lung EpiCheck software analyses the PCR output calculating an EpiScore, a numerical score (0–100) reflecting the overall methylation level in the assay's markers.

### Statistical analysis

The groups’ baseline characteristics were compared using Chi-squared test for categorical parameters and Wilcoxon's rank-sum test for continuous parameters. Sensitivity and specificity were calculated for the entire sample and for different subgroups of interest along with 95% exact binomial confidence intervals. The predictive ability of the continuous EpiScore was evaluated *via* logistic regression and the corresponding area under the receiver operator characteristic curve (AUC) was calculated. Positive likelihood ratio (LR+) and negative likelihood ratio (LR−) were calculated for the entire sample and for different subgroups of interest along with 95% exact binomial confidence intervals (LR+ = sensitivity/(1−specificity), LR− = (1−sensitivity)/specificity). A multivariable logistic regression was used to examine the relationship between the true patient status (lung cancer case or control) and their EpiScore result. The contribution of the EpiScore result was examined adjusting for the patient's personal characteristics and known risk factors for lung cancer. An additional multivariable logistic regression analysis was performed to examine whether the EpiScore outcome is affected by a patient's personal characteristics or known risk factors for lung cancer. Both analyses used the subset of patients for whom all relevant information was available. The contribution of each predictor in the model was evaluated *via* odds ratio and the overall prediction ability of the model was evaluated *via* AUC.

## Results

**FIGURE 1:**
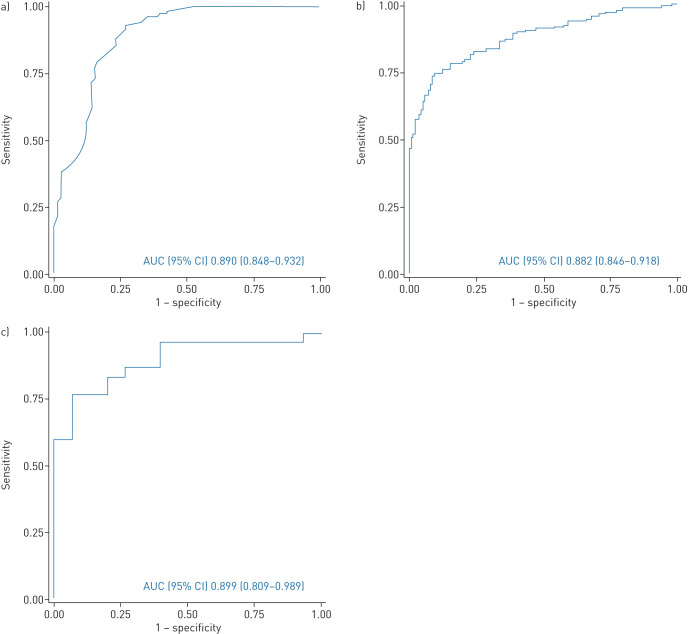
Receiver operating characteristic curves for a) training set; b) European validation set; c) Chinese validation set. AUC: area under the curve.

The training set included 367 subjects (102 cases and 265 controls). Cases were significantly older with a higher number of pack-years compared to controls, while sex and number of years since quitting smoking were similar (table 2). Smoking status was also significantly different, as half of the cases were missing this information. A balanced distribution of histological subtypes and stages was achieved with 28% of nonsmall lung cancer (NSCLC) patients having stage I disease (table 3). The AUC (95% CI) was 0.890 (0.848–0.932) (figure 1a), the sensitivity/specificity combinations were 84.3% (75.8–90.8%)/77.7% (72.2–82.6%) with LCO and 73.5% (63.9–81.8%)/93.6%(89.9–96.2%) with HCO (table 3). Likelihood ratios are reported in supplementary table S5.

**TABLE 2:** Patient demographics

	**Training set**			**European validation set**			**Chinese validation set**		
	**Cases**	**Controls**	**p-value**	**Cases**	**Control**	**p-value**	**Cases**	**Control**	**p-value**
**Subjects**	102	265		179	137		30	15	
**Age years**	67 (51–83)	62 (49–82)	p<0.0001	65 (23–89)	63 (50–87)	0.0130	64 (40–79)	33 (23–51)	p<0.0001
**Sex**			0.182			0.037			0.053
Male	70 (68.6)	162 (61.1)		132 (73.7)	86 (62.8)		21 (70.0)	6 (40.0)	
Female	32 (31.4)	103 (38.9)		47 (26.3)	51 (37.2)		9 (30.0)	9 (60.0)	
**Smoking status**			p<0.0001^§^			p<0.0001^§^			p<0.001^§^
Current smoker^#^	36 (35.3)	117 (44.2)		75 (41.9)	57 (41.6)		11 (36.7)	0 (0.0)	
Former smoker	7 (6.9)	148 (55.8)		35 (19.6)	80 (58.4)		4 (13.3)	1 (6.7)	
Never-smoker	6 (5.9)			9 (5.0)			14 (46.7)	14 (93.3)	
Unknown	53 (52.0)			60 (33.5)			1 (3.3)	0 (0.0)	
**Smoking pack-years**^¶^	40 (2–129)	20 (1–138)	0.010	41 (4–182)	20 (1–120)	p<0.0001	30 (15–80)	10 (NA)	^ƒ^
**Years since quitting smoking**^+^	13 (2–41)	17 (1–50)	0.267	18 (1–42)	17 (1–58)	0.394	7 (3–10)	10 (NA)	^ƒ^

Data are presented as n, median (range) or n (%), unless otherwise stated. ^#^: smokers reporting quitting within 1 year prior to study inclusion were considered to be current smokers; ^¶^: reported in smokers only; this information was missing for five cases in the training set; ^+^: reported in former smokers only; this information was missing for five controls in the training set, and in five patients (n=4 cases, n=1 control) in the European validation set; ^§^: this analysis included only patients in the current and former smoker categories; ^ƒ^: not calculated as there was only one control subject with history of smoking in this set.

**TABLE 3:** Lung EpiCheck performance by sensitivity and specificity

	**Training set**				**European validation set**				**Chinese validation set**			
	**LCO EpiScore=60**		**HCO EpiScore=70**		**LCO EpiScore=60**		**HCO EpiScore=70**		**LCO EpiScore=60**		**HCO EpiScore=70**	
**Cases/controls**	102/265				179/137				30/15			
**AUC (95% CI)**	0.890 (0.848–0.932)				0.882 (0.846–0.918)				0.899 (0.809–0.989)			
**Overall sensitivity**	86/102	84.3 (75.8–90.8)	75/102	73.5 (63.9–81.8)	156/179	87.2 (81.3–91.7)	133/179	74.3 (67.2–80.5)	23/30	76.7 (59.1–88.2)	17/30	56.7 (39.2–72.6)
**Overall specificity**	206/265	77.7 (72.2–82.6)	248/265	93.6 (89.9–96.2)	88/137	64.2 (55.6–72.2)	124/137	90.5 (84.3–94.9)	14/15	93.3 (68.0–99.9)	15/15	100.0 (78.1–100.0)
**Sensitivity by histological subtype**		Overall^§^ p=0.075 NSCLC *versus* SCLC p=0.213		Overall^§^ p=0.051 NSCLC *versus* SCLC p=0.278		Overall^§^ p=0.250 NSCLC *versus* SCLC p=0.224		Overall^§^ p=0.311 NSCLC *versus* SCLC p=0.120		p=0.029		p<0.001
Adenocarcinoma	34/45	75.6 (60.5–87.1)	27/45	60.0 (44.3–74.3)	73/82	89.0 (80.2–94.9)	59/82	72.0 (60.9–81.3)	12/19	63.2 (38.4–83.7)	6/19	31.6 (12.6–56.6)
Squamous cell carcinoma^#^	35/38	92.1 (78.6–98.3)	32/38	84.2 (68.7–94.0)	61/74	82.4 (71.8–90.3)	53/74	71.6 (59.9–81.5)	11/11	100.0 (71.5–100.0)	11/11	100.0 (71.5–100.0)
Other NSCLC	2/3	66.7 (9.4–99.2)	2/3	66.7 (9.4–99.2)	5/6	83.3 (35.9–99.6)	5/6	83.3 (35.9–99.6)				
All NSCLC	71/86	82.6 (72.9–89.9)	61/86	70.9 (60.1–80.2)	139/162	85.8 (79.5–90.8)	117/162	72.2 (64.7–79.0)	23/30	76.7 (57.7–90.1)	17/30	56.7 (37.4–74.5)
Small cell carcinoma	10/10	100.0 (69.2–100.0)	9/10	90.0 (55.5–99.7)	15/15	100.0 (78.2–100.0)	14/15	93.3 (68.1–99.8)				
Other/unknown	5/6	83.3 (35.9–99.6)	5/6	83.3 (35.9–99.6)	2/2	100.0 (15.8–100.0)	2/2	100.0 (15.8–100.0)				
**Sensitivity by NSCLC stage**		p=0.075		p=0.012		p=0.089		p=0.187		p=0.193		p=0.018
Stage I	18/26	69.2 (48.2–85.7)	13/26	50.0 (29.9–70.1)	29/37	78.4 (61.8–90.2)	23/37	62.2 (44.8–77.5)	7/10	70.0 (34.8–93.3)	3/10	30.0 (6.7–65.2)
Stage II	17/21	81.0 (58.1–94.6)	14/21	66.7 (43.0–85.4)	24/28	85.7 (67.3–96.0)	20/28	71.4 (51.3–86.8)	3/6	50.0 (11.8–88.2)	2/6	33.3 (4.3–77.7)
Stage III	17/20	85.0 (62.1–96.8)	16/20	80.0 (56.3–94.3)	53/59	89.8 (79.2–96.2)	44/59	74.6 (61.6–85.0)	10/11	90.9 (58.7–99.8)	10/11	90.9 (58.7–99.8)
Stage IV	17/17	100.0 (80.5–100.0)	16/17	94.1 (71.3–99.9)	33/37	89.2 (74.6–97.0)	30/37	81.1 (64.8–92.0)	3/3	100.0 (29.2–100.0)	2/3	66.7 (9.4–99.2)
Unstaged	2/2	100.0 (15.8–100.0)	2/2	100.0 (15.8–100.0)	0/1	0.0 (0.0–97.5)	0/1	0.0 (0.0–97.5)				
**Sensitivity by tumour size (largest diameter), NSCLC only**						p<0.001^ƒ^		p<0.0001^ƒ^		p=0.584		p=0.043
≤20 mm					11/16	68.8 (41.3–89.0)	7/16	43.8 (19.8–70.1)	3/5	60.0 (14.7–94.7)	1/5	20.0 (0.5–71.6)
21–30 mm					14/22	63.6 (40.7–82.8)	10/22	45.5 (24.4–67.8)	4/6	66.7 (22.3–95.7)	2/6	33.3 (4.3–77.7)
31–50 mm					46/49	93.9 (83.1–98.7)	38/49	77.6 (63.4–88.2)	11/14	78.6 (49.2–95.3)	9/14	64.3 (35.1–87.2)
>50 mm					47/50	94.0 (83.5–98.7)	44/50	88.0 (75.7–95.5)	5/5	100.0 (47.8–100.0)	5/5	100.0 (47.8–100.0)
Unknown^¶^					23/27	85.2 (66.3–95.8)	20/27	74.1 (53.7–88.9)				
**Sensitivity by tumour size (largest diameter), stage I NSCLC only**						p=0.003		p=0.020		p=0.700		p=0.200
≤20 mm					4/7	57.1 (18.4–90.1)	3/7	42.9 (9.9–81.6)	2/4	50.0 (6.8–93.2)	0/4	0.0 (0.0–60.2)
21–30 mm					6/11	54.5 (23.4–83.3)	4/11	36.4 (10.9–69.2)	3/3	100.0 (29.2–100.0)	1/3	33.3 (0.8–90.6)
>30 mm					19/19	100.0 (82.4–100.0)	16/19	84.2 (60.4–96.6)	2/3	66.7 (9.4–99.2)	2/3	66.7 (9.4–99.2)
**Sensitivity by stage group, NSCLC only**		p=0.058		p=0.008		p=0.641		p=0.465		p=0.290		p=0.196
Early stages (I, II and IIIA)	45/59	76.3 (63.4–86.4)	36/59	61.0 (47.4–73.5)	86/101	85.1 (76.7–91.4)	71/101	70.3 (60.4–79.0)	17/24	70.8 (48.9–87.4)	12/24	50.0 (29.1–70.9)
Advanced stages (IIIB and IV)^+^	24/25	96.0 (79.6–99.9)	23/25	92.0 (74.0–99.0)	53/60	88.3 (77.4–95.2)	46/60	76.7 (64.0–86.6)	6/6	100.0 (54.0–100.0)	5/6	83.3 (35.9–99.6)
**Sensitivity by SCLC stage**		p=1.000		p=0.300		p=1.000		p=1.000				
Limited	3/3	100.0 (29.2–100.0)	2/3	66.7 (9.4–99.2)	6/6	100.0 (54.1–100.0)	6/6	100.0 (54.1–100.0)				
Extensive	7/7	100.0 (59.0–100.0)	7/7	100.0 (59.0–100.0)	9/9	100.0 (66.4–100.0)	8/9	88.9 (51.8–99.7)				

Data are presented as n/N or % (95% CI), unless otherwise stated. LCO: low cut-off; HCO: high cut-off; AUC: area under the curve; NSCLC: nonsmall cell lung cancer; SCLC: small cell lung cancer. In the cases of complete or quasi-complete separation, exact p-values were calculated. ^#^: patients with adenosquamous carcinoma histology were grouped with the squamous cell carcinoma histology subtype; ^¶^: tumour sizes were missing or unmeasurable for n=3 stage II, n=14 stage III and n=10 stage IV; ^+^: stage III tumours without stage IIIA/IIIB classification were included in the advanced stages group; ^§^: p-value calculation for the comparison of histological subtypes did not include the “unknown” group; ^ƒ^: p-value calculation for the comparison by tumour size did not include the “unknown” group.

The European validation set included 363 subjects, out of which 316 subjects were eligible and had EpiScore results (179 cases and 137 controls; supplementary figure S3a). Cases were heavier smokers (median 41 *versus* 20 pack-years), slightly older (median age 65 *versus* 63 years) and were more likely to be male compared to controls (74% *versus* 63%); all statistically significant (table 2). Despite similar rates of current smokers (42%), smoking status was significantly different, as a third of the cases were missing this information. Years since quitting smoking were similar between the groups (18 *versus* 17 years). Adenocarcinoma was the most common histological subtype (46%), followed by squamous cell carcinoma (41%) (table 3). Small cell lung cancer (SCLC) was underrepresented compared to the incidence reported in the literature (8% *versus* 13% [12]). A balanced distribution of NSCLC stages was achieved and 26% of NSCLC patients had stage I disease (table 3). The AUC was 0.882 (0.846–0.918) (figure 1b) overall and 0.797 (0.704–0.889), 0.830 (0.764–0.895) and 0.862 (0.813–0.910) in stage I, stages I and II and early stages (stages I, II and IIIA), respectively (supplementary figure S4). Overall sensitivity/specificity combinations were 87.2% (81.3–91.7%)/64.2% (55.6–72.2%) with LCO and 74.3% (67.2–80.5%)/90.5% (84.3–94.9%) with HCO. Applying the LCO/HCO cut-offs, Lung EpiCheck detected 85.1% (76.7–91.4%)/70.3% (60.4–79.0%) of early-stage NSCLC, 78.4% (61.8–90.2%)/62.2% (44.8–77.5%) of stage I NSCLC and 57.1% (18.4–90.1%)/42.9% (9.9–81.6%) of stage I NSCLC ≤20 mm. Lung EpiCheck demonstrated high sensitivity of 100.0% (54.1–100.0%)/100.0% (54.1–100.0%) in limited SCLC and 100.0% (66.4–100.0%)/88.9% (51.8–99.7%) in extensive SCLC with LCO/HCO; however, the numbers of SCLC were small. Sensitivities were similar (p>0.05) across histological subtypes, NSCLC stages, NSCLC early-/late-stage groups and limited/extensive SCLC for each cut-off. The only factor significantly impacting sensitivity in univariate analyses were tumour size (p<0.001/p<0.0001 in LCO/HCO) and tumour size of stage I (p=0.003/p=0.020).

**FIGURE 2:**
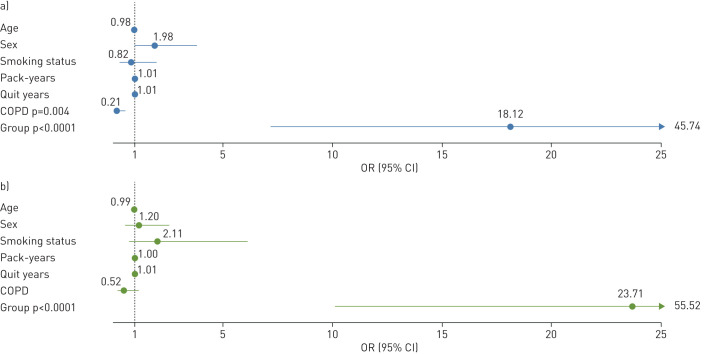
Multivariable logistic regression of factors potentially impacting Lung EpiCheck positive result, by cut-off: a) low cut-off EpiScore ≥60; b) high cut-off EpiScore ≥70. This analysis included only patients with history of smoking and full smoking data, n=242 (n=106 cases, n=136 controls). Risk factors included age, pack-years and quit years as continuous measures; sex (female *versus* male), smoking status (former *versus* current smoker), COPD (yes *versus* no), and group (cases *versus* controls). For current smokers, quit years were counted as 0.

In a multivariable analysis of patients with smoking information (n=242), established risk factors for lung cancer (age, smoking status, pack-years and quit years) and sex did not influence having a positive Lung EpiCheck result in either cut-off (figure 2). Presence of COPD significantly decreased the chance of having a positive Lung EpiCheck result at LCO. A trend of higher EpiScores in patients without COPD *versus* patients with the condition was maintained when looking at various statistical measures of EpiScore (mean, median, 1st and 3rd quartile) of cases and in controls separately, but when combining the two groups, this trend was reversed (data not shown). The only factor driving a positive result was the group (case/control) with odds ratio (95% CI) of 18.2 (7.2–45.7), p<0.0001 with LCO and 23.7 (10.1–55.5), p<0.0001 with HCO. Likelihood ratios are reported in supplementary table S5.

**FIGURE 3:**
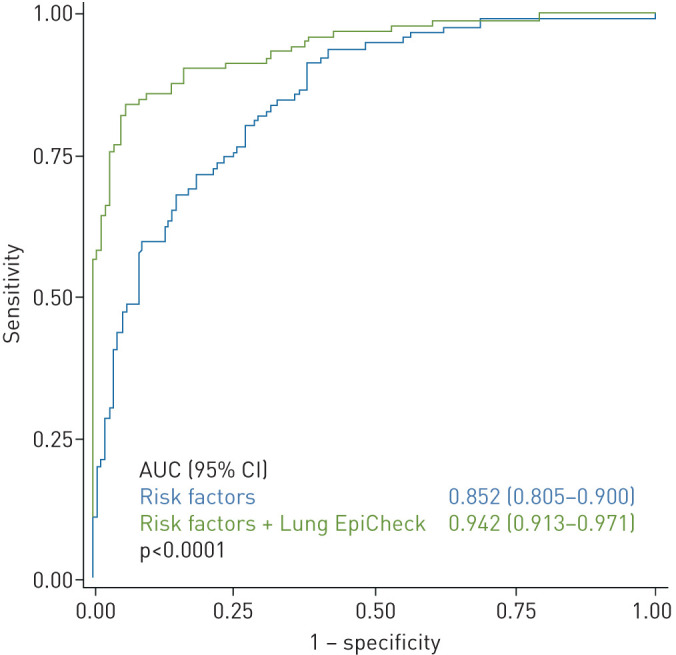
Multivariable logistic regression analysis of predictive factors for lung cancer. This analysis included only patients with history of smoking and full smoking data, n=242 (n=106 cases, n=136 controls). Risk factors: age, pack-years, quit years (continuous), sex (male/female), smoking status (current/past), COPD (yes/no). For current smokers, quit years were counted as 0. AUC: area under the curve.

A multivariable analysis was performed to assess the accuracy of lung cancer prediction based on risk factors alone, or in combination with EpiScore (figure 3). In our data, age, sex, smoking status, quit years, pack-years and COPD together yielded an AUC (95% CI) of 0.852 (0.805–0.900). Adding EpiScore significantly increased the AUC to 0.942 (0.913–0.971), p<0.0001. This analysis was performed on a subset of 242 patients with full smoking history, and the Lung EpiCheck AUC was similar to that of the entire set (0.881 (0.843–0.918) *versus* 0.882 (0.846–0.918)), which suggests that this subsample is representative of the entire set; however, these results should be interpreted with caution.

10 (3.1%) of the tests in the validation sets failed to yield a result; two due to insufficient amount of DNA in the sample, and eight due to failed plasma separation.

The Chinese validation set enrolled 92 sequential cases and 15 controls. 41 cases were eligible, out of which 30 were selected to ensure good representation of all stages (n=10 stage I, n=6 stage II, n=11 stage III and n=3 stage IV) (supplementary figure S3b). As expected, age and female/male ratio were not comparable between cases and controls, as the latter were healthy volunteers (table 2). The AUC was 0.899 (0.809–0.989) (figure 1c), the sensitivity/specificity combinations were 76.7% (59.1–88.2%)/93.3% (68.0–99.9%), respectively, with LCO and 56.7% (39.2–72.6%)/100.0% (78.1–100.0%), respectively, with HCO (table 3). Lung EpiCheck with LCO/HCO detected 70.8% (48.9–87.4%)/50% (29.1–70.9%) of early-stage cancers, 70% (34.8–93.3%)/30% (6.7–65.2%) of stage I cancers and 60% (14.7–94.7%)/20% (0.5–71.6%) of stage I NSCLC ≤20 mm. Likelihood ratios are reported in supplementary table S5.

No tests failed in the Chinese set.

## Discussion

Our data demonstrate that Lung EpiCheck achieved performance characteristics suggesting that, if prospectively validated, may be suitable for clinical use in early detection of lung cancer. The predictive performance of Lung EpiCheck in the European validation data was very high, with AUC of 0.882. While maximising sensitivity (87.2%) with the LCO, the specificity remained good (64.2%), and while maximising specificity (90.5%) using the HCO, the sensitivity remained high (74.3%). In the Chinese set the AUC of 0.899 yielded high specificity in both cut-offs (LCO 93.3% and HCO 100.0%), with good sensitivity with LCO (76.7%). Differences between the validation sets are probably due to including young nonsmoking controls and surgical patients with small resectable tumours in the Chinese set. Detection at early stages is the key performance factor for Lung EpiCheck to ensure patients are detected in time for curative treatment. In the European set, the AUCs were consistently high in stage I NSCLC (0.797), stages I and II NSCLC (0.830) and early-stage (stages I, II and IIIA) NSCLC (0.862). In the validation sets, Lung EpiCheck detected 70–80% of stage I NSCLC with LCO, detecting tumours as small as 8 mm (adenocarcinoma in the Chinese set). With HCO, European results remained strong with stage I NSCLC sensitivity of 62.2%, but Chinese performance deteriorated to 30.0%. Early-stage performance should be interpreted with caution, as the controls were not scanned or followed-up to ensure no asymptomatic cancer existed. These results compare favourably with published results of other blood tests for lung cancer detection, reporting stage I sensitivity ∼40% (22–71%), many of which are from training sets [13–17]. Sensitivity of NSCLC tumours was significantly impacted by size, but even in stage I NSCLC ≤20 mm, there is a good signal of effectiveness detecting ≥50% of tumours with LCO (four out of seven and two out of four in the European and Chinese validations sets, respectively). With HCO this stage I NSCLC ≤20 mm sensitivity was similar in the European set (three out of seven), but all were missed in the Chinese set. Likelihood ratios can be used to simply and quickly estimate the post-test probability; however, there is no established gold-standard threshold for determining an acceptable likelihood ratio in the developing field of biomarkers for lung cancer screening. Regardless, we believe that the likelihood ratios achieved by Lung EpiCheck appear to be in a good range.

Both the Centers for Medicare & Medicaid Services [18] and the U.S. Preventive Services Task Force [19] recommend lung cancer screening with LDCT for high-risk populations, but national screening rates are very low, up to 14% [5]. Obstacles to lung cancer screening uptake are probably due to patient and primary care provider concerns about costs, inconvenience and possible risks associated with radiation and false-positive results. Additional limiting factors are absence of efficient proven programmes or lack of programme infrastructure. Offering a simple blood test to noncompliant eligible patients, as a tool to motivate them to get LDCT, could help overcome some of these barriers. Ease and safety of a blood test could encourage patients to be tested, and a positive blood test result could potentially convince patients to participate in LDCT screening programmes. If performance is confirmed in a prospective clinical study, prioritising eligible patients for LDCT based on such a test could alleviate systems restraints by reducing the number of unnecessary procedures and providing effective patient selection. Reducing the number of LDCTs could also indirectly impact on the number of false-positive findings, and their adverse outcomes and costs.

Alternatively, such a test can be used to better identify high-risk people who should undergo LDCT. Currently, high-risk populations are defined by demographic and exposure factors (age and smoking history) with very limited discrimination of AUC 0.6–0.7 [20]. Subsequently, in the USA, a mere 2.51 cancers are detected per 1000 LDCT scans [21] and >50% of lung cancer patients will not be considered eligible [7]. More elaborate risk models, such as PLCOm2012 (Prostate, Lung, Colorectal and Ovarian Cancer Screening Trial), report better performance (AUC 0.7–0.8) as they include other surrogate markers such as COPD and history of cancer, but they are more cumbersome and harder to implement in the clinical routine. In our analysis, relationship of cases and positive Lung EpiCheck did not vary substantially by value of other risk factors (age, pack-years, quit years, sex, smoking status and COPD). Presence of these risk factors did not change or impact the strong relationship between lung cancer and the test result. Moreover, combining Lung EpiCheck with risk factors achieved a very high discrimination of 94.2%, allowing for optimal selection of high-risk populations for lung cancer screening. Further validation is required to confirm Lung EpiCheck predictive performance and to define the best way to combine Lung EpiCheck and risk factors.

Similar to published evidence, showing correlation of cfDNA levels in the blood with tumour burden from NSCLC [22] and other solid tumours [23–26], Lung EpiCheck sensitivity correlated with tumour size. Two studies found cfDNA signal to inversely correlate with survival in patients with newly diagnosed lung cancer [27, 28], suggesting that it is also a prognostic marker for aggressiveness of the tumour. Further investigations are needed to inform whether there is a lower size limit of detectability by cfDNA, and if lack of cfDNA signal is an independent prognostic factor, or potentially a sign of overdiagnosis. Either way, a liquid biopsy for early detection must be very sensitive, in order to pick up the signal in the blood of early curable cancers. Lung EpiCheck's good preliminary results in early cancer classification can be explained by an analytical sensitivity of 1:200 000 [29] which is 20–200-fold higher than other liquid biopsy products available [30–32].

Cost-effectiveness is an important consideration in the applicability of screening tests. In a recently published model, in order to maintain the cost-effectiveness threshold of USD 50 000 per life-year gained, a marker added to risk factors to improve selection of patients for lung cancer screening could cost up to USD 300 [33]. Therefore, the next-generation sequencing based liquid biopsy tests, common in advanced settings, are irrelevant for this field, as their running costs alone are currently much higher at USD 1000–2000 per test. Similarly, other available lung cancer biomarker tests have sensitivity and specificity below screening requirements, lowering further the price level they can charge to be cost-effective. In contrast, Lung EpiCheck, with its high preliminary performance and its simple PCR-based technology, appear to be potentially well situated to be cost effective and commercially viable.

Mutations are established key factors of cancer development (*e.g.* driver mutations, acquired therapy resistance) as well as important targets for treatment, and could be potential markers for lung cancer detection. However, mutations in genes such as p53 [34] drive clonal haematopoiesis [35] in up to 21% of healthy elderly people. This can pose as a serious confounder and can generate false-positive results when using these mutations in blood tests for early detection of cancer. Alternatively, and unhampered by such problems, methylation changes have recently emerged as promising markers for cancer detection [16, 36].

### Limitations

Case–control studies are prone to selection bias, as cases and controls do not necessarily come from the same population and are not truly comparable. This is reflected in our study by the significant difference between the groups in parameters such as age, sex, smoking status and pack-years. Cases were patients diagnosed with lung cancer due to any reason (symptoms, incidental finding, screening), and do not reflect a screening population. Controls did not receive LDCT screening, nor were they followed-up after blood draw; therefore, it is not known whether lung cancer cases were among them and were missed. Therefore, a prospective study in high-risk individuals undergoing LDCT with follow-up for lung cancer incidence is essential to confirm the current study findings. Staging was performed locally according to local standard of care in each site, so in the European validation there was a mix between AJCC7 and AJCC8. This probably translates to a potential overlap between large stage I to small stage II cancers. Collection of some data were limited in biobank samples, leading to missing smoking histories in 19% of the European validation set, limiting the multivariable analyses to a subpopulation of that set. Personal history of cancer is a known risk factor for lung cancer [37]; however, to ensure that the signal emerges from lung cancer, such patients were excluded. The Chinese set was a small single-centre study that included surgical patients only, therefore not representative of the Chinese lung cancer population; additionally, the controls were young, healthy and mostly nonsmokers, not representative of patients at risk. A larger prospective study in China is warranted to confirm the performance of Lung EpiCheck in this population.

Our current findings need to be validated in prospective trials.

### Conclusions

Lung EpiCheck demonstrated strong suggestive performance in lung cancer prediction in case–control European and Chinese samples, detecting up to 78% of stage I tumours, up to 100% of SCLC and significantly improving predictive accuracy when added to established risk factors. Prospective studies are required to confirm these findings. Utilising such a simple and inexpensive blood test to select people for lung cancer screening has the potential to improve compliance and broaden access to screening for high-risk populations.

## Supplementary material

10.1183/13993003.02682-2020.Supp1 **Please note:** supplementary material is not edited by the Editorial Office, and is uploaded as it has been supplied by the author.
Supplementary material ERJ-02682-2020.Supplement

## Shareable PDF

10.1183/13993003.02682-2020.Shareable1 This one-page PDF can be shared freely online.
Shareable PDF ERJ-02682-2020.Shareable
