# Supplementary material for: Validation of Lung EpiCheck, a novel methylation-based blood assay, for the detection of lung cancer in European and Chinese high-risk individuals
Source: Eur Respir J. 2021 Jan 14;57(1):2002682. doi: 10.1183/13993003.02682-2020 (PMC7806969; doi:10.1183/13993003.02682-2020)
Supplement: Supplementary file 1 [file ERJ-02682-2020.Supplement.pdf]

Validation of Lung EpiCheck®, a novel methylation-based blood assay, for the detection of lung cancer in European and Chinese high-risk individuals

SUPPLEMENTARY MATERIALS

Supplemental Figure S1. Marker panel and algorithm development

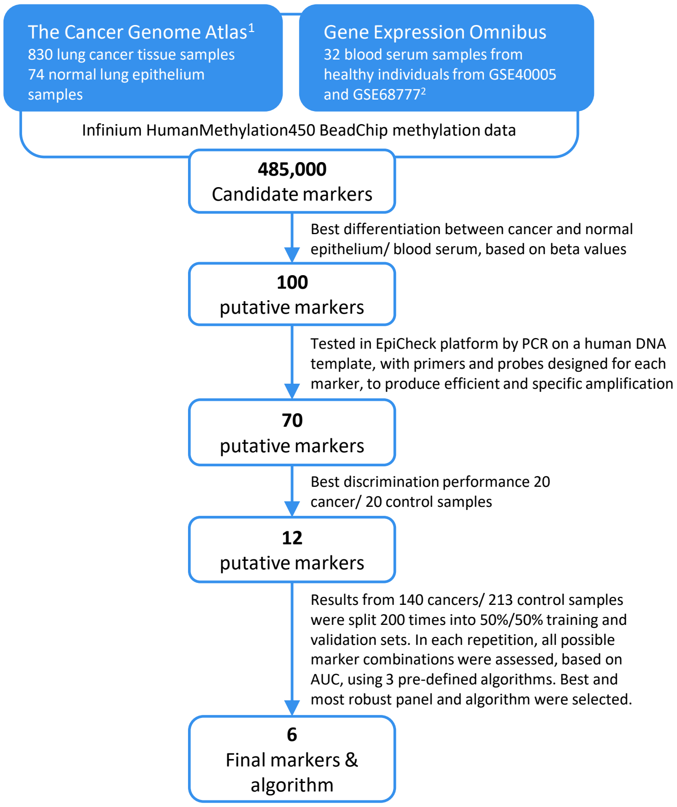

1 Weisenberger. Characterizing DNA methylation alterations from The Cancer Genome Atlas. J Clin Investig 2014;124:17–23
2 Sabunciyan et al. Association of DNA Methylation with Acute Mania and Inflammatory Markers. PLoS One 2015;10(7):e0132001

Supplemental Table S2. List of sites, European sets

1	Athens Chest Hospital Sotiria, Athens, Greece
2	BioIVT (Seralab) LLC.
3	Carmel Medical Center, Haifa, Israel
4	Emek Medical Center, Afula, Israel
5	Holy Family Hospital, Nazareth, Israel
6	Indivumed GmBH
7	Maccabi Petach Tikva Clinic, Maccabi Health services, Petach Tikva, Israel
8	Maccabi Ramat Hasharon Clinic, Maccabi Health services, Ramat Hasharon, Israel
9	Meir Medical Center, Kfar Saba, Israel
10	National Institute of Tuberculosis and Lung Diseases, Warsaw, Poland
11	National Korányi Institute of Pulmonology, Budapest, Hungary
12	Shaare Zedek Medical Center, Jerusalem, Israel
13	Shamir Medical Center (Assaf Harofeh), Zrifin, Israel
14	Shuali Ra’anana Clinic, Clalit Health Services, Ra’anana, Israel
15	Soroka Medical Center, Beer Sheva, Israel
16	Tel-Aviv Sourasky Medical Center (Ichilov), Tel-Aviv, Israel
17	Tissue solutions Ltd.
18	Wolfson Medical Center, Holon, Israel
19	Ziekenhuisgroep Twente (ZGT), Hengelo, the Netherlands
20	Ziv Medical Center, Safed, Israel

Supplemental Figure S3. CONSORT diagrams

S3a. CONSORT diagram European validation set

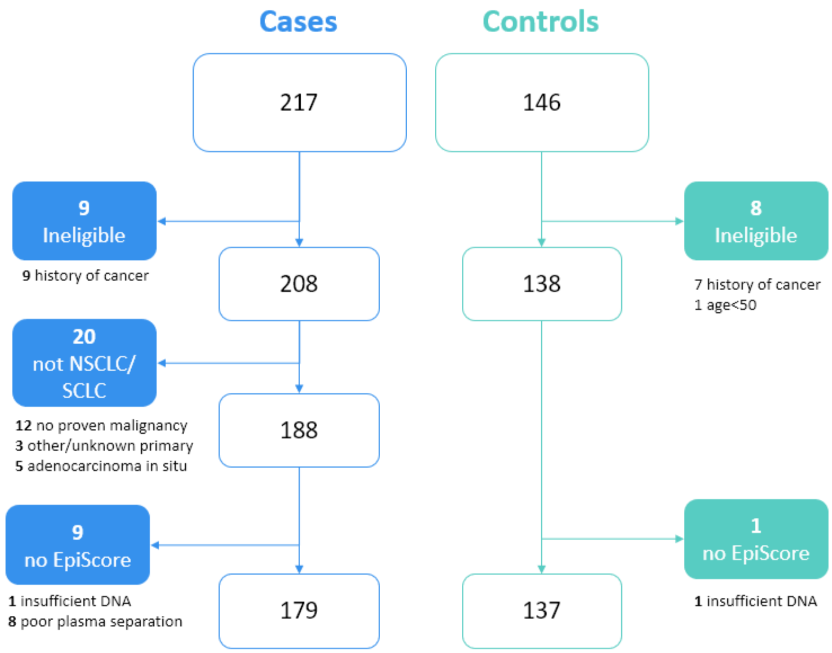

S3.b. CONSORT diagram Chinese validation set

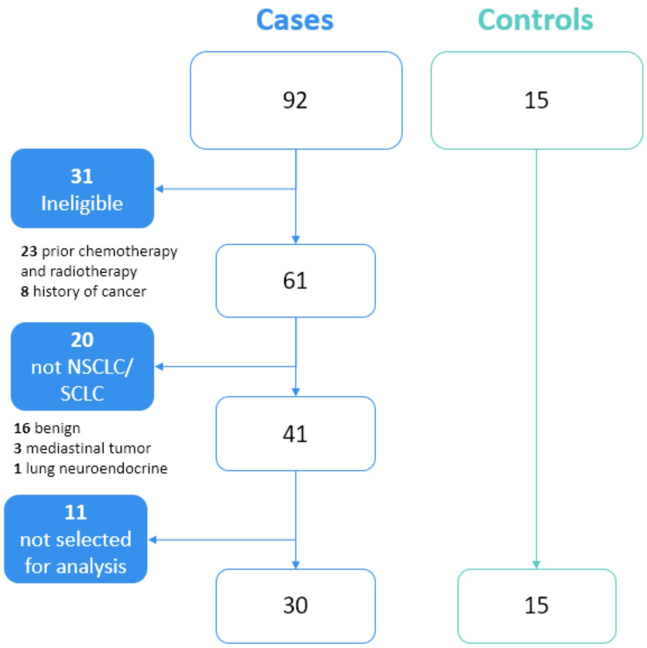

Supplemental Figure S4. ROC curves by extent of disease

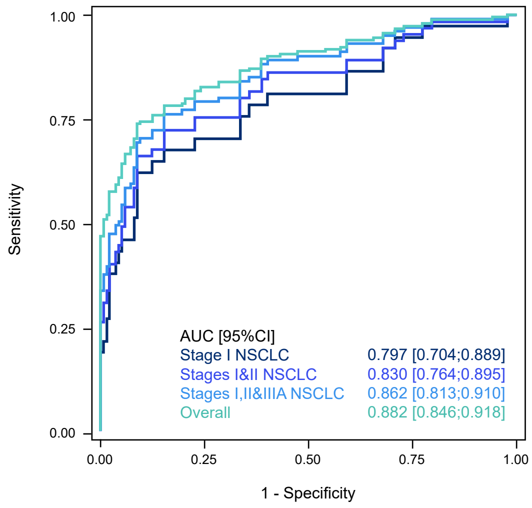

Supplemental Table S5. Lung EpiCheck performance by likelihood ratio

	Training Set		European Validation Set		Chinese Validation Set	
	Low cut-off EpiScore=60	High cut-off EpiScore=70	Low cut-off EpiScore=60	High cut-off EpiScore=70	Low cut-off EpiScore=60	High cut-off EpiScore=70
**Positive Likelihood Ratio [95% CI]**	3.8 [3.0;4.8]	11.5 [7.1;18.4]	2.4 [1.9;3.1]	7.8 [4.6;13.2]	11.5 [1.7;77.2]	18.1 [1.2;281.4]
**Negative Likelihood Ratio [95% CI]**	0.2 [0.1;0.3]	0.3 [0.2;0.4]	0.2 [0.1;0.3]	0.3 [0.2;0.4]	0.2 [0.1;0.5]	0.4 [0.3;0.7]
**Positive Likelihood Ratio by histological subtype [95% CI]**						
Adenocarcinoma	3.4 [2.6;4.5]	9.4 [5.6;15.7]	2.5 [2.0;3.2]	7.6 [4.4;12.9]	9.5 [1.4;64.9]	10.4 [0.6;171.1]
Squamous Cell Carcinoma	4.1 [3.2;5.3]	13.1 [8.1;21.2]	2.3 [1.8;3.0]	7.5 [4.4;12.9]	15.0 [2.3;99.6]	30.7 [2.0;470.6]*
Other NSCLC	3.0 [1.3;6.9]	10.4 [4.1;26.2]	2.3 [1.5;3.6]	8.8 [4.7;16.5]		
All NSCLC	3.7 [2.9;4.7]	11.1 [6.8;17.9]	2.4 [1.9;3.0]	7.6 [4.5;12.9]	11.5 [1.7;77.2]	18.1 [1.2;281.4]
Small cell carcinoma	4.5 [3.6;5.6]	14.0 [8.5;23.2]	2.8 [2.2;3.5]	9.8 [5.8;16.8]		
Other/ Unknown	3.7 [2.5;5.7]	13.0 [7.3;23.3]	2.8 [2.2;3.5]	10.5 [6.3;17.7]		
**Positive Likelihood Ratio by NSCLC stage**						
I	3.1 [2.2;4.4]	7.8 [4.3;14.2]	2.2 [1.7;2.9]	6.6 [3.7;11.6]	10.5 [1.5;72.8]	10.2 [0.6;178.2]
II	3.6 [2.7;4.9]	10.4 [6.0;18.0]	2.4 [1.8;3.1]	7.5 [4.3;13.3]	7.5 [1.0;58.6]	11.4 [0.6;208.5]
III	3.8 [2.9;5.1]	12.5 [7.5;20.8]	2.5 [2.0;3.2]	7.9 [4.6;13.5]	13.6 [2.0;91.4]	28.0 [1.8;432.2]
IV	4.5 [3.6;5.6]	14.7 [9.1;23.6]	2.5 [1.9;3.2]	8.5 [5.0;14.7]	15.0 [2.3;99.6]	20.0 [1.2;339.5]
Unstaged	4.5 [3.6;5.6]	15.6 [9.8;24.7]	0.7 [0.1;7.8]	2.6 [0.2;30.7]		
**Positive Likelihood Ratio by tumor size (largest diameter), NSCLC only**						
≤20	1.8 [0.8;4.0]	4.7 [1.6;13.4]	1.9 [1.3;2.9]	4.6 [2.2;9.8]	9.0 [1.2;68.1]	8.0 [0.4;170.6]
21-30	3.1 [2.0;4.8]	7.2 [3.4;15.2]	1.8 [1.2;2.6]	4.8 [2.4;9.6]	10.0 [1.4;72.2]	11.4 [0.6;208.5]
31-50	3.9 [3.0;5.2]	12.3 [7.5;20.4]	2.6 [2.1;3.3]	8.2 [4.8;14.0]	11.8 [1.7;79.8]	20.3 [1.3;318.7]
>50	4.5 [3.6;5.6]	15.6 [9.8;24.7]	2.6 [2.1;3.3]	9.3 [5.5;15.7]	15.0 [2.3;99.6]	29.3 [1.9;453.8]*
Unknown	4.1 [3.2;5.2]	12.1 [7.3;19.8]	2.4 [1.8;3.1]	7.8 [4.4;13.7]		
**Positive Likelihood Ratio by tumor size (largest diameter), stage I NSCLC only**						
≤20	0.6 [0.1;4.0]	1.0 [0.1;14.8]	1.6 [0.8;3.2]	4.5 [1.7;12.3]	7.5 [0.9;63.2]	3.2 [0.1;141.5]**
21-30	3.5 [2.3;5.3]	6.9 [2.9;16.4]	1.5 [0.9;2.7]	3.8 [1.5;9.8]	15.0 [2.3;99.6]	12.0 [0.6;242.8]
>30	4.5 [3.6;5.6]	13.9 [8.3;23.2]	2.8 [2.2;3.5]	8.9 [5.1;15.4]	10.0 [1.3;78.1]	20.0 [1.2;339.5]
**Positive Likelihood Ratio by stage group, NSCLC only**						
Early stages (I,II & IIIA)	3.4 [2.6;4.5]	9.5 [5.8;15.7]	2.4 [1.9;3.0]	7.4 [4.3;12.6]	10.6 [1.6;71.8]	16.0 [1.0;251.8]
Advanced stages (IIIB, IIIC, IV, IVA)	4.3 [3.4;5.5]	14.3 [8.9;23.0]	2.5 [1.9;3.1]	8.1 [4.7;13.8]	15.0 [2.3;99.6]	25.1 [1.6;395.5]
**Positive Likelihood Ratio by SCLC stage**						
Extensive	4.5 [3.6;5.6]	15.6 [9.8;24.7]	2.8 [2.2;3.5]	9.5 [5.4;16.6]		
Limited	4.5 [3.6;5.6]	10.4 [4.1;26.2]	2.8 [2.2;3.5]	10.5 [6.3;17.7]		

For all tumor characteristics, only Positive Likelihood Ratio [95% CI] is presented. The denominator for these PLRs is the overall (1-specificity) of each set.

*Positive Likelihood Ratio was adjusted as sensitivity was 100%

**Positive Likelihood Ratio was adjusted as sensitivity was
