# Supplementary material for: Validation of Lung EpiCheck, a novel methylation-based blood assay, for the detection of lung cancer in European and Chinese high-risk individuals
Source: Eur Respir J. 2021 Jan 14;57(1):2002682. doi: 10.1183/13993003.02682-2020 (PMC7806969; doi:10.1183/13993003.02682-2020)
Supplement: Supplementary file 2 [file ERJ-02682-2020.Shareable.pdf]

ORIGINAL ARTICLE
LUNG CANCER

SHAREABLE PDF

Validation of Lung EpiCheck, a novel methylation-based blood assay, for the detection of lung cancer in European and Chinese high-risk individuals

Mina Gaga[1], Joanna Chorostowska-Wynimko[2], Ildikó Horváth[3], Martin C. Tammemagi[4], David Shitrit[5], Vered H. Eisenberg[6], Hao Liang[7], David Stav[8], Dan Levy Faber[9,10], Maarten Jansen[11], Yael Raviv[12], Vasileios Panagoulias[13], Piotr Rudzinski[2], Gabriel Izbicki[14], Ohad Ronen[15], Adiv Goldhaber[16], Rawia Moalem[17], Nadir Arber[18], Ilana Haas[19] and Qinghua Zhou[7]

Affiliations: [1]7th Respiratory Medicine Dept, Athens Chest Hospital, Athens, Greece. [2]National Institute of Tuberculosis and Lung Diseases, Warsaw, Poland. [3]National Korányi Institute of Pulmonology, Budapest, Hungary. [4]Brock University, St Catharines, ON, Canada. [5]Pulmonary Dept, Meir Medical Center, Kfar Saba, Israel. [6]Sackler Faculty of Medicine, Tel Aviv University, Tel Aviv, Israel. [7]Lung Cancer Center/Lung Cancer Institute, West China Hospital, Sichuan University, Chengdu, China. [8]Lung Institute, Maccabi Health Services Hashalom, Tel-Aviv, Israel. [9]Dept of Cardiothoracic Surgery, Lady Davis Carmel Medical Center, Haifa, Israel. [10]Ruth and Bruce Rappaport Faculty of Medicine, Technion – Israel Institute of Technology, Haifa, Israel. [11]Pulmonary Dept, Ziekenhuisgroep Twente, Hengelo, The Netherlands. [12]Dept of Medicine, Pulmonology Institute, Soroka Medical Center, Ben-Gurion University, Beer-Sheva, Israel. [13]2nd Respiratory Medicine Dept, Athens Chest Hospital, Athens, Greece. [14]Pulmonary Institute, Shaare Zedek Medical Center, Jerusalem, Israel. [15]Dept of Otolaryngology – Head and Neck Surgery, Galilee Medical Center affiliated with Azrieli Faculty of Medicine, Safed, Israel. [16]Clalit Health Services, Raanana, Israel. [17]Gastroenterology Institute, The Holy Family Hospital, Nazareth, Israel. [18]Integrated Cancer Prevention Center, Tel Aviv Sourasky Medical Centre, Sackler Faculty of Medicine, Tel Aviv University, Tel-Aviv, Israel. [19]Breast Unit, Meir Medical Center, Kfar Saba, Israel.

Correspondence: Mina Gaga, 7th Resp. Med. Dept and Asthma Center, 152 Mesogion Ave Athens 11527, Greece. E-mail: minagaga@yahoo.com

@ERSpublications
Lung EpiCheck, a simple blood test, detected 85% of early-stage lung cancers with specificity of 64% in high-risk population, reaching AUC of 0.942 when combined with risk factors. This could improve efficiency of implementing lung cancer screening. https://bit.ly/3jWhLOn

Cite this article as: Gaga M, Chorostowska-Wynimko J, Horváth I, *et al*. Validation of Lung EpiCheck, a novel methylation-based blood assay, for the detection of lung cancer in European and Chinese high-risk individuals. *Eur Respir J* 2021; 57: 2002682 [https://doi.org/10.1183/13993003.02682-2020].

This single-page version can be shared freely online.

ABSTRACT

Aim: Lung cancer screening reduces mortality. We aim to validate the performance of Lung EpiCheck, a six-marker panel methylation-based plasma test, in the detection of lung cancer in European and Chinese samples.

Methods: A case–control European training set (n=102 lung cancer cases, n=265 controls) was used to define the panel and algorithm. Two cut-offs were selected, low cut-off (LCO) for high sensitivity and high cut-off (HCO) for high specificity. The performance was validated in case–control European and Chinese validation sets (cases/controls 179/137 and 30/15, respectively).

Results: The European and Chinese validation sets achieved AUCs of 0.882 and 0.899, respectively. The

Copyright ©ERS 2021. This version is distributed under the terms of the Creative Commons Attribution Non-Commercial Licence 4.0.

sensitivities/specificities with LCO were 87.2%/64.2% and 76.7%/93.3%, and with HCO they were 74.3%/90.5% and 56.7%/100.0%, respectively. Stage I nonsmall cell lung cancer (NSCLC) sensitivity in European and Chinese samples with LCO was 78.4% and 70.0% and with HCO was 62.2% and 30.0%, respectively. Small cell lung cancer (SCLC) was represented only in the European set and sensitivities with LCO and HCO were 100.0% and 93.3%, respectively. In multivariable analyses of the European validation set, the assay's ability to predict lung cancer was independent of established risk factors (age, smoking, COPD), and overall AUC was 0.942.

Conclusions: Lung EpiCheck demonstrated strong performance in lung cancer prediction in case–control European and Chinese samples, detecting high proportions of early-stage NSCLC and SCLC and significantly improving predictive accuracy when added to established risk factors. Prospective studies are required to confirm these findings. Utilising such a simple and inexpensive blood test has the potential to improve compliance and broaden access to screening for at-risk populations.
